# Psychological factors and consumer behavior during the COVID-19 pandemic

**DOI:** 10.1371/journal.pone.0256095

**Published:** 2021-08-16

**Authors:** Adolfo Di Crosta, Irene Ceccato, Daniela Marchetti, Pasquale La Malva, Roberta Maiella, Loreta Cannito, Mario Cipi, Nicola Mammarella, Riccardo Palumbo, Maria Cristina Verrocchio, Rocco Palumbo, Alberto Di Domenico

**Affiliations:** 1 Department of Neuroscience, Imaging and Clinical Sciences, G. d’Annunzio University of Chieti-Pescara, Chieti, Italy; 2 Department of Psychological, Health and Territorial Sciences, G. d’Annunzio University of Chieti-Pescara, Chieti, Italy; 3 Center for Advanced Studies and Technology (CAST), G. d’Annunzio University of Chieti-Pescara, Chieti, Italy; 4 Department of Business Studies, Grenon School of Business, Assumption University, Worcester, MA, United States of America; University of Hradec Kralove: Univerzita Hradec Kralove, CZECH REPUBLIC

## Abstract

The COVID-19 pandemic is far more than a health crisis: it has unpredictably changed our whole way of life. As suggested by the analysis of economic data on sales, this dramatic scenario has also heavily impacted individuals’ spending levels. To better understand these changes, the present study focused on consumer behavior and its psychological antecedents. Previous studies found that crises differently affect people’s willingness to buy necessities products (i.e., utilitarian shopping) and non-necessities products (i.e., hedonic shopping). Therefore, in examining whether changes in spending levels were associated with changes in consumer behavior, we adopted a fine-grained approach disentangling between necessities and non-necessities. We administered an online survey to 3833 participants (age range 18–64) during the first peak period of the contagion in Italy. Consumer behavior toward necessities was predicted by anxiety and COVID-related fear, whereas consumer behavior toward non-necessities was predicted by depression. Furthermore, consumer behavior toward necessities and non-necessities was predicted by personality traits, perceived economic stability, and self-justifications for purchasing. The present study extended our understanding of consumer behavior changes during the COVID-19 pandemic. Results could be helpful to develop marketing strategies that consider psychological factors to meet actual consumers’ needs and feelings.

## Introduction

Coronavirus disease 2019 (COVID-19) refers to an infection (SARS-CoV-2) of the lower respiratory tract [1, 2], which was first detected in Wuhan (China) in late December 2019. Since then, the number of contagions by COVID-19 has been increasing globally each day [3]. In March 2020, the World Health Organization (WHO) declared the COVID-19 outbreak a global pandemic [4]. Subsequently, several national governments implemented long-term full or partial lockdown measures to reduce the spread of the virus. Although these strict measures have proven to be quite effective in containing the further spread of the virus, they have severely impacted the global economic system and caused an unprecedented shock on economies and labor markets [5]. As a matter of fact, the COVID-19 pandemic can be defined as far more than just a health crisis since it has heavily affected societies and economies. COVID-19 outbreak has unpredictably changed how we work, communicate, and shop, more than any other disruption in this decade [6]. As reflected by the analysis of economic data on sales, this dramatic situation has greatly influenced consumer attitudes and behaviors. According to a study conducted by the Nielsen Company, the spread of the COVID-19 pandemic led to a globally manifested change in spending levels related to consumer behavior [7]. Specifically, a growing tendency in the sales of necessities has been observed: consumer priorities have become centered on the most basic needs, including food, hygiene, and cleaning products. In Italy, consumer shopping preferences have changed throughout the pandemic. Initially, when Italy was the first country in Europe to experience the spreading of COVID-19 (between March and April 2020). Consumer behavior tended to compulsively focus on purchasing essential goods, especially connected with preventing the virus, such as protective devices and sanitizing gel [8]. The pandemic changed the consumption patterns, for instance reducing sales for some product categories (e.g., clothes), and improving sales for other categories (e.g., entertainment products) [9]. Also, research indicated that job insecurity and life uncertainty experienced during the pandemic negatively impacted on consumer behavior of Italian workers [10].

It comes as no surprise that in such a situation of emergency, the need for buying necessities takes precedence [11]. However, the investigation of antecedent psychological factors, including attitudes, feelings, and behaviors underlying changes in consumer behavior during the COVID-19 pandemic, have received less attention. Nevertheless, understanding the psychological factors which drive consumer behavior and products choices can represent a crucial element for two main reasons. First, such investigation can extend our understanding of the underpinnings of the changes in consumer behavior in the unprecedented context of COVID-19. Second, obtained results could be helpful in the development of new marketing strategies that consider psychological factors to meet actual consumers’ needs and feelings [12]. On the one side, companies could benefit from this knowledge to increase sales during the COVID-19 pandemic [13]. Moreover, understanding these needs and feelings could be fundamental to improve the market’s preparedness to face future pandemics and emergencies [14, 15]. On the other hand, consumers could take advantage of this new market’s preparedness to respond to their actual needs and feelings. As a result, in case of future emergency, factors such as anxiety and a perceived shortage of essential goods could be reduced [16], whereas well-being and the positive sense of self of the consumers could be supported [17]. Furthermore, the novelty of the present study lies in two main aspects. First, based on previous studies highlighting that crises differently affect people’s willingness to buy necessities and non-necessities products [11, 18], we adopted a fine-grained approach and disentangled between necessities and non-necessities. Second, considering the unprecedented context of the COVID-19 pandemic, we adopted an integrative approach to investigate the role of different psychological factors such as fear, anxiety, stress, depression, self-justifications, personality traits, and perceived economic stability in influencing consumer behavior. Noteworthy, all these factors have been implicated in consumer behavior in previous research, but, to our knowledge, no study has considered all of them at once. Therefore, considering both the lack of studies that have focused on these factors at once and the unique opportunity to study them in the context of such an unprecedented global pandemic, we adopted an integrative approach to get one of the first overviews of the role of the several psychological factors influencing consumer behavior.

Previous studies in consumer psychology and behavioral economics have highlighted that several psychological factors impact consumer behavior differently [18–20]. Consumer behavior refers to the study of individuals or groups who are in the process of searching to purchase, use, evaluate, and dispose of products and services to satisfy their needs [12]. Importantly, it also includes studying the consumer’s emotional, mental, and behavioral responses that precede or follow these processes [21]. Changes in consumer behavior can occur for different reasons, including personal, economic, psychological, contextual, and social factors. However, in dramatic contexts such as a disease outbreak or a natural disaster, some factors, more than others, have a more significant impact on consumer behavior. Indeed, situations that potentially disrupt social lives, or threaten individuals’ health, have been proven to lead to strong behavioral changes [22]. An example is panic buying, a phenomenon occurring when fear and panic influence behavior, leading people to buy more things than usual [23]. Specifically, panic buying has been defined as a herd behavior that occurs when consumers buy a considerable amount of products in anticipation of, during, or after a disaster [24]. A recent review on the psychological causes of panic buying highlighted that similar changes in consumer behavior occur when purchase decisions are impaired by negative emotions such as fear and anxiety [25]. Noteworthy, in the context of the COVID-19 pandemic, Lins and Aquino [23] showed that panic buying was positively correlated with impulse buying, which has been defined as a complex buying behavior in which the rapidity of the decision process precludes thoughtful and deliberate consideration of alternative information and choice [25]. The analysis of the different psychological factors involved in consumer behavior and changes in purchase decisions still represents an area that is scarcely explored. Arguably, during an uncertain threatening situation, such as a health crisis or a pandemic, the primitive part of our brain usually becomes more prominent, pushing individuals to engage in behaviors that are (perceived as) necessary for survival [26–29]. Importantly, these primitive instinctual behaviors can override the rational decision-making process, having an immense impact on usual consumer behavior. Therefore, the basic primitive response of humans represents the core factor responsible for changes in consumer behavior during a health crisis [16]. Specifically, fear and anxiety originated from perceived feelings of insecurity and instability, are the factors driving these behavioral changes [30]. In line with the terror management theory [31], previous studies have shown that external events, which threaten the safety of individuals, motivate compensatory response processes to alleviate fear and anxiety [32, 33]. These response processes can prompt individuals to make purchases to gain a sense of security, comfort, and momentarily escape, which can also serve as a compensatory mechanism to alleviate stress. However, as such buying motivation represents an attempt to regulate the individuals’ negative emotions, the actual need for the purchased products is often irrelevant [34].

Pandemics and natural disasters are highly stressful situations, which can easily induce negative emotions and adverse mental health states [35–37] such as perceived lack of control and instability, which are core aspects of emergency situations, contribute directly to stress. In turn, research has highlighted that stress is a crucial factor in influencing consumer behavior. For example, past studies have shown that individuals may withdraw and become passive in response to stress, and this inaction response can lead to a decrease in purchasing [38, 39]. However, some studies point out that stress can lead to an active response, increasing impulsive spending behaviors [40, 41]. Moreover, event-induced stress can lead to depressive mood. In some cases, the depressive mood may translate into the development of dysfunctional consumer behavior, such as impulsive (the sudden desire to buy something accompanied by excessive emotional response) and/or compulsive buying (repetitive purchasing due to the impossibility to control the urge) [41, 42]. In this context, Sneath and colleagues [37] highlighted that changes in consumer behavior often represent self-protective strategies aimed at managing depressive states and negative emotions by restoring a positive sense of self. Importantly, a recent study conducted during the COVID-19 pandemic showed that depression predicted the phenomenon of the over-purchasing, which was framed as the degree to which people had increased their purchases of some necessities goods (e.g. food, water, sanitary products, pharmacy products, etc.) because of the pandemic [43].

A recent study recommended a differentiation between necessity and non-necessity products to better understand consumer behavior in response to stressful situations [18]. According to the authors, contrasting findings on the link between stress and consumer behavior may be due to the fact that stress affects certain purchasing behaviors negatively, but others positively, depending on the type of product under investigation. On one side, it has been argued that consumers may be more willing to spend money on necessities (vs. non-necessities) by making daily survival products readily available. Accordingly, recent research documented an increase in buying necessities products (i.e., utilitarian shopping) during and after a traumatic event [11]. However, other findings showed that impulsive non-necessities purchasing (i.e., hedonic shopping) could also increase as an attempt to escape or minimize the pain for the situation. That is, non-necessities buying is used as an emotional coping strategy to manage stress and negative emotional states [44]. To reconcile these findings, Durante and Laran [18] proposed that people adopt strategic consumer behavior to restore their sense of control in stressful situations. Hence, high stress levels generally lead consumers to save money and spend strategically on products perceived as necessities. Importantly, regarding the impact of perceived stress due to the COVID-19 pandemic on consumer behavior, a recent study showed that the likelihood of purchasing quantities of food larger than usual increased with higher levels of perceived stress [45].

Another psychological factor implicated in consumer behavior that deserves special attention is self-justification strategies [46]. Self-justification refers to the cognitive reappraisal process by which people try to reduce the cognitive dissonance stemming from a contradiction between beliefs, values, and behaviors. People often try to justify their decisions to avoid the feeling of being wrong to maintain a positive sense of self [17]. In consumer behavior research, it is widely acknowledged that consumers enhance positive arguments that support their choices and downplay counterarguments that put their behavior in question [47]. Based on previous research, it is plausible that, within the context of the COVID-19 pandemic, self-justifications for buying non-necessities products may also include pursuing freedom and defying boredom [11, 48]. Further, the hedonistic attitude of “I could die tomorrow” or “You only live once” could certainly see a resurgence during the COVID-19 emergency [48], and become a crucial mechanism accounting for individual differences in consumer behavior. Based on these considerations, in the context of the COVID-19 pandemic, self-justifications strategies could be relevant for non-necessities, since products for fun or entertainment could be more suited to the pursuit of freedom and to defy boredom. Conversely, self-justifications strategies related to necessities could be implemented to a lesser degree, due to the very nature of the products. The unprecedented context of the pandemic could already justify the purchase of those essential goods by itself, and additional justifications may not be necessary.

Furthermore, several studies have shown that household income has a significant impact in determining people’s expenses [49–51]. Not surprisingly, the research highlighted a positive relationship between income and spending levels [52]. Income is defined as money received regularly from work or investments. Interestingly, a different line of research pointed out that self-perceived economic stability is a more appropriate determinant of consumer behavior than actual income [53, 54]. Usually, people tend to report subjective feelings of income inadequacy, even when their objective financial situation might not support such attitude [55]. An interesting explanation for this bias draws on the social comparison process. Indeed, the study of Karlsson et colleagues [53] showed that, compared to families who considered themselves to have a good financial situation, households which considered themselves to be worse off economically than others reported fewer purchases of goods, perceived the impact of their latest purchase on their finance to be greater, and planned purchases more carefully. Furthermore, a recent study in the context of the COVID-19 emergency showed that people who believed to have limited financial resources were the most worried about the future [56, 57]. Therefore, in the present study, we measured both the income and the perceived economic situation of the respondents to respectively consider the objective economic information and the subjective perception of respondents. However, considering the state of uncertainty experienced by many households during the COVID-19 pandemic [58], we changed the comparison from other families to participants’ economic situation in different time frames. We asked respondents to report perceived economic stability before, during, and after the emergency.

Finally, besides situational factors related to the specific emergency, the individuals’ personality traits are likely to have a role in determining consumer behavior as well. Past research has highlighted that the Big Five personality traits [59] can differently predict consumer behavior [60]. Specifically, conscientiousness, openness, and emotional stability (alias neuroticism) were related to compulsive buying, impulsive buying, and utilitarian shopping. Nevertheless, how different personality traits are related to consumer behavior is still an open question [61].

**Fig 1:**
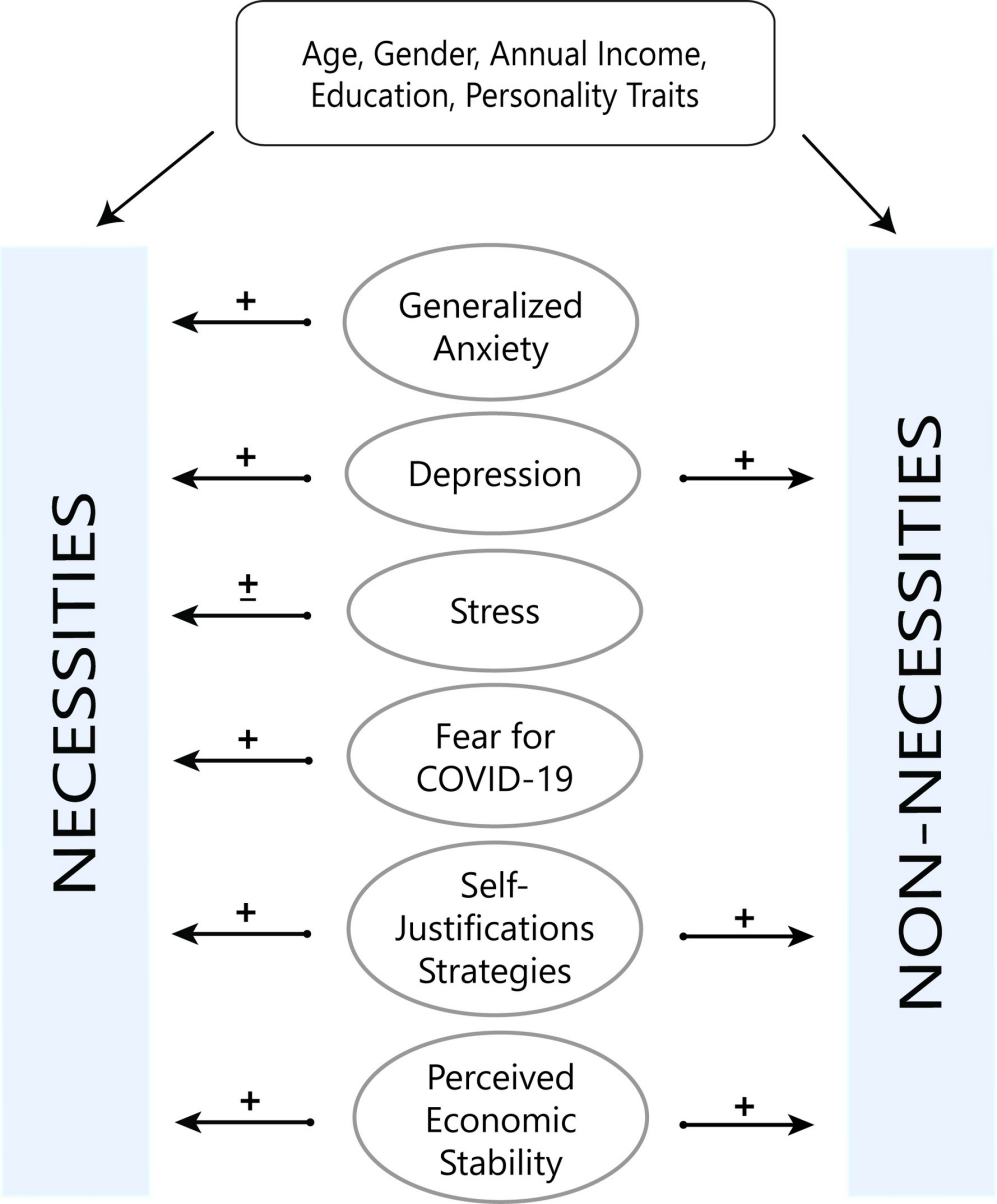
Conceptual model of the different mutual relationships between the constructs involved in the study.

We conducted a nationwide survey in the Italian population to examine consumer behavior during the lockdown phase due to the COVID-19 pandemic. Since the COVID-19 emergency has emphasized the usefulness of essential goods (e.g. food, medications, etc.) compared to non-essential products (e.g. luxury items such as clothes and accessories) [62], in our study, we categorized products in necessities and non-necessities. Furthermore, changes in spending levels (necessities vs. non-necessities) were examined to confirm the effect that COVID-19 had on people’s expenses. Moreover, we tried to clarify the relationship between changes in spending levels and changes in consumer behavior. Finally, we focused on the psychological factors underlying changes in consumer behavior toward the target products. Based on the literature, we expected to find an increase in purchases with a more noticeable rise in necessity products. Specifically, we explored potential underpinnings of consumer behavior by examining mood states and affective response to the emergency, perceived economic stability, self-justification for purchasing, and personality traits. All these factors have been implicated in consumer behavior in previous research, but, to our knowledge, no study has considered all of them at once. Therefore, in this study, we adopted an integrative approach to study the contribution of different psychological factors by considering their mutual influence (see Fig 1). Specifically, based on the empirical findings and theoretical accounts presented above, we hypothesized that during the COVID-19 pandemic:

- Higher levels of anxiety and COVID-related fear would explain changes in consumer behavior, increasing the need for buying necessities.
- Higher levels of stress would lead consumers to save money or, in alternative, would increase the need to spend money on necessities (i.e., utilitarian shopping).
- Higher levels of depressive state would be associated with an increase in the need for buying, both necessities and non-necessities.

- Higher implementation of self-justification strategies would be associated with a higher need for buying, especially for non-necessities.
- Higher perceived economic stability would be associated with an increase in the need for both necessities and non-necessities.

The construct involved in the study is placed in the center of the figure. Arrows depart from these constructs to show the hypothesized relationship between the constructs and the outcomes of the present study (Necessities and Non-necessities). The symbol “±” was used to take into consideration two possible opposite directions.

## Materials and methods

Data were collected through a series of questionnaires, using a web-based survey implemented on the Qualtrics software. The survey was active in the period starting from April 1st, 2020, to April 20th, 2020, during the first peak of the contagion in Italy. We used a convenience sample due to the exceptional situation of the COVID-19 pandemic and the time constraints to conduct our investigation. Therefore, participants were recruited through word-of-mouth and social media. Inclusion criteria were the age over 18 and be resident in Italy. First, socio-demographic information was collected, including gender, age, annual income, and education. Then, questions on spending levels and consumer behavior, both before the COVID-19 pandemic and during the first week of lockdown in Italy, were presented, separating necessities and non-necessities. Finally, a series of specifically created questionnaires and standardized measures were administered to investigate psychological and economic variables.

### Participants

A total of 4121 participants were initially recruited. For the present study, we adopted a rigorous approach, excluding 104 participants over the age of 64, since they relied on retirement benefits and -from an economic point of view- were considered a specific population, not comparable to the rest of the sample [63]. Furthermore, we excluded 184 participants who did not report spending any money before the COVID-19 pandemic on buying necessities and/or non-necessities. Therefore, 3833 Italian participants (69.3% women, age *M* = 34.2, *SD* = 12.5) were included in this study. All participants provided their written informed consent before completing the survey. The study was conducted following the ethical standards of the Declaration of Helsinki and was approved by the Institutional Review Board of Psychology (IRBP) of the Department of Psychological, Health and Territorial Sciences at G. d’Annunzio University of Chieti-Pescara (protocol number: 20004). Participants did not receive monetary or any other forms of compensation for their participation.

### Demographic variables

A demographic questionnaire was administered to collect background information. The questions considered age, gender, annual income, and education. The annual income was then categorized into five levels, based on the income brackets established by the Italian National Statistical Institute [64]. Education was categorized into five levels, from elementary to school to postgraduate degree.

### Consumer behavior during COVID-19

We created this questionnaire from scratch to get a comprehensive overview of people’s economic attitudes and behaviors during the COVID-19 emergency. The idea of this new questionnaire was developed based on a series of previous studies on consumer behavior [43, 65–67]. However, specific items were developed from scratch adapting them to the specific unprecedented context of the COVID-19 pandemic. Specifically, these items were created following a series of group discussions between all co-authors of the present study. To directly measure changes in consumer behavior due to the COVID-19 pandemic, participants were requested to compare their actual behavior to their normal behavior before the COVID-19 outbreak. Therefore, the initial statement in the questionnaire underlined that answers had to be given by referring to the COVID-19 emergency period compared to everyday life before the outbreak.

The factor structure and reliability were evaluated in the larger sample (*n* = 4121), using principal component analysis (PCA) and Cronbach’s alpha. The results revealed a six-factor structure and satisfactory reliability values (see S1 Table for more details). Note that the PCA and reliability analyses were also conducted on the current subsample, and the pattern of results did not change.

For the present study’s aims, we focused on three scales: “Necessities”, “Non-necessities”, and “Self-justifications”. Items are shown in Table 1. The first two scales investigated consumer behavior toward the different framed products. Specifically, items addressed the individual’s attitudes, feelings, and behaviors toward necessities and non-necessities. Thus, higher scores reflected greater value (e.g., need, utility) placed on the target products.

**Table 1:** Questionnaires’ items.

***Consumer behavior during COVID-19" questionnaire***	
**Scale**	**Item**
*Necessities*	1. I have felt the need to buy larger amounts of health and safety products (e.g., alcohol-based hand sanitizer, gloves, face masks)
	2. I believe I impulsively bought Necessities products
	3. I have felt the need to buy larger amounts of Necessities products (e.g., food, health and wellness products, personal hygiene products, house cleaning products) compared to before
	4. If you do not already own health and safety products, how much more would you be willing to spend to acquire these products (e.g. alcohol-based hand sanitizer, gloves, face masks)?
	5. I felt the need to buy products that I did not need before
*Non-necessities*	1. At the time of purchase, how useful did you think Non-necessities products would be?
	2. At this time, how useful do you think the Non-necessities products that you purchased are?
	3. Have you bought any products that are considered Non-necessities?
	4. I have felt the need to buy larger amounts of Non-necessities products (e.g., products for fun and entertainment) compared to before
*Self-justifications*	1. Making purchases makes me feel better
	2.Considering that I am restricted to home isolation, I want to enjoy the purchases that I have made
	3. Considering that I am saving money by not going out, I can afford to make a new purchase
	4. I am happy with the purchases that I have made
*Spending habits*	1.Before COVID-19 emergency, how much do you think you were spending weekly for Necessities products?
	2. During COVID-19 emergency, how much do you think you have spent weekly for Necessities products?
	3. Before COVID-19 emergency, how much do you think you were spending weekly for Non-necessities products?
	4. During COVID-19 emergency, how much do you think you have spent weekly for Non-necessities products?
***Fear for COVID-19***	
**Scale**	**Item**
*Belief of contagion*	1. I often thought I was infected with the virus
	2. I think I could be infected with the virus in the future
	3. I think that a dear or close person to me could potentially be infected with the virus
	4. I think that a dear or close person to me could potentially be infected with the virus in the future
*Consequences of contagion*	1. I think that a person infected with the virus could recover
	2. I think that a person infected with the virus could die
	3. I think it is probable that I would recover after being infected with the virus
	4. I think that being infected with the virus could be lethal for me

*Note*. Items referred to the COVID-19 emergency period compared to before COVID-19 outbreak.

The self-justifications scale referred to consumers’ thoughts to justify their purchases, with no distinction between necessity and non-necessity products. Higher scores reflected a frequent use of self-justifications in purchasing items.

For all these scales, responses were given on a Likert scale ranging from 0 (*not at all*), to 100, (*extremely*). Total scores on each scale were obtained by averaging all items.

### Change in spending levels due to COVID-19

A fourth scale, i.e. “Spending Habits,” was extracted from the questionnaire mentioned above. As we aimed at measuring changes in the spending levels due to the COVID-19 emergency, we decided to use single items instead of the total scale score (items are presented in Table 1). Specifically, we created three percentage scores: “Changes in General Spending”, “Changes in Necessities spending”, and “Changes in Non-necessities spending” considering the difference between the money spent during the first week of lockdown, and the money spent on average in a week before the emergency (see Table 1 notes). Scores reflect the change in the amount (in Euro) that people devolved in purchasing the target products (hypothetical range from -1999 to +1999).

### Big Five Inventory 10-item (BFI-10)

Big Five Inventory 10-item (BFI-10) is a short scale designed to briefly assess the five personality traits with two items for each trait. Specifically, these traits are: Agreeableness (example item: “I see myself as someone who is generally trusting”), Conscientiousness (example item: “I see myself as someone who does a thorough job”), Emotional stability (example item: “I see myself as someone who is relaxed, handles stress well”), Extraversion (example item: “I see myself as someone who is outgoing, sociable”), and Openness (example item: “I see myself as someone who has an active imagination”) [68]. In addition, respondents are asked to indicate whether they agree or disagree with each statement on a 5-point Likert-type scale, ranging from 1 (*not agree at all*) to 5 (*totally agree*). A previously validated Italian version was used in the present study [69].

### Generalized anxiety disorder (GAD-7)

The GAD-7 [70] is a 7-item self-reported measure designed to screen for generalized anxiety disorder and to measure the severity of symptoms, based on the DSM-IV criteria. This measure is often used in both clinical practice and research. Specifically, respondents are asked the frequency they have experienced anxiety symptoms in the past two weeks (e.g., “Not being able to stop or control worrying”) on a 4-point Likert scale, ranging from 0 (*not at all*) to 3 (*nearly every day*). The total score ranges from 0 to 21, with higher scores indicating worse anxiety symptomatology.

### Patient health questionnaire (PHQ-9)

The patient health questionnaire (PHQ-9) is a 9-item self-reported brief diagnostic measure for depression [71]. Specifically, respondents are asked of the frequency they felt bothered by several depressive symptoms during the past two weeks (e.g., “Little interest or pleasure in doing things”) on a 4-point Likert scale, ranging from 0 (*not at all*) to 3 (*nearly every day*). Total score ranges from 0 to 27, with higher scores indicating higher depressive symptoms.

### Perceived Stress Scale (PSS)

The Perceived Stress Scale (PSS) is a 14-item self-report measure designed to assess the degree to which situations are appraised as stressful [72]. Each item (e.g., “In the last month, how often have you been upset because of something that happened unexpectedly?”) is rated on a 5-point Likert scale ranging from 0 (*never*) to 4 (*very often*). Thus, the total score ranges from 0 to 56, with a higher score indicating a higher level of perceived stress during the COVID-19 emergency.

### Fear for COVID-19

We administered the Fear for COVID-19 questionnaire to measure fear and concerning beliefs related to the COVID-19 pandemic [35, 36, 73]. This questionnaire was created from the assumption that, during a health crisis, the individual’s fear is determined by both the hypothesized susceptibility (i.e., probability of contracting a disease) and the expected severity of the event (i.e., perceived consequences of being infected) [25]. Therefore, the 8 items dealt with the perceived probability of being infected by COVID-19 (Belief of contagion) and the possible consequences of the contagion (Consequences of contagion). See Table 1 for the complete list of the items. Previous studies have reported the PCA and reliability of the questionnaire [36]. Responses were given on a Likert scale ranging from 0 (*not at all*), to 100, (*extremely*). A total score was obtained by averaging the items (range 0–100).

### Perceived economic stability

This questionnaire was developed to assess the subjective perception of an individual’s economic situation. The PCA in the larger sample revealed a unidimensional structure (see S2 Table for more details). The scale assessed perceived economic stability in three different timepoints: before, during, and after (in terms of expectation) the COVID-19 pandemic. Responses were given on a Likert scale ranging from 0 (*not at all*), to 100, (*extremely*). The total score was calculated by averaging these three items (range 0–100).

### Statistical analysis

We preliminary investigated changes in spending levels due to the COVID-19 pandemic, comparing expenses before the emergency to expenses during the COVID-19 pandemic. First, we analyzed changes in the average general spending level. Then, we performed dependent (paired) sample *t*-tests between “Changes in necessities spending” and “Changes in non-necessities spending” to examine differences between products framed as necessities and non-necessities.

Afterward, we checked whether changes in spending levels were associated with changes in consumer behavior by conducting Pearson’s correlation analyses, respectively between “Changes in necessities spending” and “Necessities”, and “Changes in non-necessities spending” and “Non-necessities” scores.

Finally, to investigate the psychological underpinnings of consumer behavior, we performed two hierarchical multiple regressions, respectively, with “Necessities” (Model 1) and “Non-necessities” (Model 2) as outcomes. The same predictors were entered in Model 1 and Model 2. Specifically, the order of the steps was designed to include at first the socio-demographic information as control variables. Hence, we entered the age, gender, annual income brackets, and education in the first step. In Step 2, we included the personality measures (i.e., Big-Five personality traits) since these traits are stable and are not affected by the specific situation. In Step 3, Anxiety, Depression, and Stress were entered, to analyze the impact of emotional antecedents of consumer. Further, we decided to include Fear for the COVID-19 in a separate fourth step to evaluate the effect of this specific aspect. We included perceived economic stability at Step 5 after the psychological variables. This choice allowed to analyze the impact of the perceived economic stability after controlling for the role of emotional antecedents on consumer behavior. Finally, following the same logic, we included self-justifications strategies.

## Results

Considering “Changes in General spending”, our results showed that our sample reported, on average, an increase of 60.48% in the general spending level during the first week of lockdown. Furthermore, significant differences between “Changes in Necessities spending” and “Changes in Non-necessities spending”, *t*(3832) = 11.99, *p* < .001, were detected. Indeed, the spending level for necessities products showed an increase of 90.69%, while for non-necessities products, the average increase was only 36.11%. Means and standard deviations are presented in Table 2.

**Table 2:** Means and standard deviation of variables in the study.

Variable	Mean	SD
Changes in General spending	60.48	156.20
Changes in Necessities spending	36.11	246.12
Changes in Non-necessities spending	90.69	199.84
Necessities	44.78	19.95
Non-necessities	26.55	21.19
BFI-10 - Agreeableness	6.43	1.65
BFI-10 - Conscientiousness	7.53	1.65
BFI-10 - Emotional Stability	6.04	2.10
BFI-10 - Extraversion	6.14	1.79
BFI-10 - Openness	7.09	1.90
GAD-7	7.17	4.48
PHQ-9	7.60	4.78
PSS	23.83	8.03
Fear for COVID-19	43.22	19.37
Perceived economic stability	60.58	24.75
Self-Justifications	38.58	22.83

*Note*. The Changes in General spending score was obtained from the Spending Habits scale using the following formula: (Item 2 + Item 4)—(Item 1 + Item 3)/(Item 1 + Item 3)*100. The Changes in Necessities spending score was obtained from the Spending Habits scale using the following formula: (Item 2—Item 1)/Item 1*100. The Changes in Non-necessities spending score was obtained from the Spending Habits scale using the following formula: (Item 4—Item 3)/Item 3*100. BFI-10 = The Big Five Inventory 10-item. GAD-7 = Generalized anxiety disorder. PHQ-9 = Patient Health Questionnaire. PSS = Perceived Stress Scale.

The results of the correlation analyses indicated that there was a significant positive association between “Changes in necessities spending” and “Necessities”, *r*(3831) = .22, *p* < .001. Furthermore, a significant positive association was highlighted between “Changes in non-necessities spending” and “Non-necessities”, *r*(3831) = .23, *p* < .001. Therefore, people’s changes in spending levels were related to their attitudes and feelings toward specific products. This finding supported our choice to investigate the psychological underpinnings of people’s consumer behavior.

Hierarchical multiple regression analyses were performed on the two consumer behavior scores. In addition, control variables, psychological factors, and economic variables were entered as predictors as detailed above.

Regarding Model 1 (Necessities), results showed that all the steps explained a significant amount of additional variance (see Table 3 for detailed results). When personality traits were entered in the model (Step 2), only agreeableness, openness, and emotional stability negatively predicted the outcome. However, when anxiety, depression, and stress were entered in the model (Step 3), only openness remained statistically significant. The variables entered in Step 3 contributed to explaining 7% of the variance, with anxiety and stress positively predicting the outcome. Adding fear for COVID-19 in the following step increased the explained variance by 6%, reduced the impact of anxiety, and completely overrode the effect of stress, which became non-significant. In the following steps, perceived economic stability offered a small but significant contribution (1%), and Self-justifications explained even further variance (4%). Overall, in the final step, the final model explained 23% of the variance in Necessities. Inspecting coefficients, we found that, after accounting for control variables, openness (*p* < .001), anxiety (*p* < .001), fear for COVID-19 (*p* < .001), perceived economic stability (*p* < .001), and self-justifications (*p* < .001) emerged as significant predictors.

**Table 3:** Summary of regression analysis for variables predicting "Necessities" (Model 1).

Variable	Step 1				Step 2				Step 3				Step 4				Step 5				Step 6			
	*B*	*SE*	β	*t*	*B*	*SE*	β	*t*	*B*	*SE*	β	*t*	*B*	*SE*	β	*t*	*B*	*SE*	β	*t*	*B*	*SE*	β	*t*
Age	-.11	.03	-.07	**-4.27** ***	-.07	.03	-.04	**-2.48** *	-.04	.03	-.02	**-**1.44	-.07	.03	-.04	**-2.63** **	-.05	.03	-.03	**-1.99** *	-.01	.03	.00	0.21
Gender	3.30	.70	.08	**4.71** ***	1.57	.71	.04	**2.22** *	-.45	.69	-.01	-0.65	-.77	.67	-.02	-1.14	-.44	.67	-.01	-0.66	.57	.66	.01	0.87
Income Brackets	1.04	.26	.06	**3.96** ***	1.02	.26	.06	**3.97** ***	1.02	.25	-.06	**4.11** ***	.91	.24	.06	**3.78** ***	.54	.25	.03	**2.22** *	.63	.24	.04	**2.63** **
Education	-.84	.41	-.03	**-2.05** *	-.09	.41	-.00	**-0.23**	.65	.40	.03	**1.63**	.48	.38	.02	**1.25**	.27	.38	.01	**0.71**	.00	.37	.00	**0.01**
BFI-10 - Agreeableness					-.53	.20	-.04	**-2.65** **	-.31	.19	-.03	-1.62	-.24	.19	-.02	-1.27	-.26	.19	-.02	-1.40	-.17	.18	-.01	-0.92
BFI-10 - Conscientiousness					-.33	.20	-.03	-1.62	-.03	.20	-.00	-0.16	.06	.19	.01	0.31	.16	.19	.01	0.81	.31	.19	.03	1.64
BFI-10 - Emotional Stability					-1.66	.16	-.18	**-10.32** ***	-.20	.18	-.02	-1.11	-.21	.17	-.02	-1.22	-.20	.17	-.02	-1.14	-.16	.17	-.02	-0.97
BFI-10 - Extraversion					.16	.18	.01	0.91	.18	.17	.02	1.02	.14	.17	.01	0.84	.14	.17	.01	0.85	.10	.16	.01	0.63
BFI-10 - Openness					-.50	.17	-.05	**-2.96** **	-.64	.16	-.06	**-3.97** ***	-.57	.16	-.05	**-3.55** **	-.57	.16	-.05	**-3.65** ***	-.70	.15	-.07	**-4.62** ***
GAD-7									1.08	.10	.24	**10.56** ***	.86	.10	.19	**8.66** ***	.89	.10	.20	**8.94** ***	.92	.10	.21	**9.52** ***
PHQ-9									.14	.10	.03	1.41	.05	.10	.01	0.54	.09	.10	.02	0.94	.08	.10	.02	0.79
PSS									.20	.06	.08	**3.30** ***	.05	.06	.02	0.86	.08	.06	.03	1.44	.08	.06	.03	1.46
Fear for COVID-19													.27	.02	.26	**15.93** ***	.27	.02	.26	**16.05** ***	.23	.02	.23	**14.08** ***
Economic Stability																	.09	.01	.11	**7.35** ***	.06	.01	.07	**4.62** ***
Self-justifications																					.19	.01	.22	**14.40** ***
*R* ^*2*^	.02				.05				.12				.18				.19				.23			
*R* ^*2*^ *Change*	.02				.04				.07				.06				.01				.04			
*F for change in R* ^*2*^	14.45***				30.18***				98.99***				253.58***				54.07***				207.39***			

*Note*. BFI-10 = The Big Five Inventory 10-item. GAD-7 = Generalized anxiety disorder. PHQ-9 = Patient Health Questionnaire. PSS = Perceived Stress Scale.

*p < .05,

**p < .01,

***p < .001.

In Model 2 (Non-necessities), results indicated that each step significantly contributed to explaining the outcome (see Table 4). In Step 2, personality traits explained 2% of the outcome variance, with consciousness and openness emerging as significant predictors and remaining significant until the final step. Notably, consciousness was negatively associated with non-necessities behavior, while high scores in openness were associated with higher scores on the Non-necessities scale. In Step 3, only depression was significantly and positively related to the outcome and remained so in subsequent models. Both fear for COVID-19 and perceived economic stability further significantly explained the outcome, albeit weakly (about 1% of variance each one). Higher levels of fear and perceived economic stability were associated with higher scores on the Non-necessities scale. Noteworthy, adding Self-justifications in the final step explained a substantial share of variance, equal to 12%. Specifically, higher scores on self-justifications were associated with higher scores on the Non-necessities scale. Furthermore, self-justifications also had a greater impact on non-necessities compared to those had on necessities, *t* (7664) = -10.60, *p* < .05. Total variance explained in the final step was 22%, with conscientiousness (*p* < .001), openness (*p* = .001), depression (*p* = .002), perceived economic stability (*p* = .009), and self-justifications (*p* < .001) being significant predictors.

**Table 4:** Summary of regression analysis for variables predicting "Non-necessities" (Model 2).

Variable	Step 1				Step 2				Step 3				Step 4				Step 5				Step 6			
	*B*	*SE*	β	*t*	*B*	*SE*	β	*t*	*B*	*SE*	β	*t*	*B*	*SE*	β	*t*	*B*	*SE*	β	*t*	*B*	*SE*	β	*t*
Age	-40	.03	-.24	**-15.25** ***	-.34	.03	-.20	**-12.40** ***	-.32	.03	-.19	**-11.30** ***	-.33	.03	-.20	**-11.56** ***	-.31	.03	-.18	**-11.00** ***	-.21	.03	-.13	**-7.96** ***
Gender	-5.04	.72	-.11	**-6.99** ***	-4.80	.74	-.10	**-6.52** ***	-5.24	.75	-.11	**-7.02** ***	-5.32	.75	-.12	**-7.14** ***	-4.99	.74	-.11	**-6.73** ***	-3.21	.70	-.07	**-4.61** ***
Income Brackets	.50	.27	.03	1.84	.51	.27	.03	1.91	.54	.27	.03	**2.00** *	.51	.27	.03	1.89	.14	.27	.01	0.51	.29	.25	.02	1.13
Education	2.65	.42	.10	**6.28** *	2.65	.42	.10	**6.26** ***	2.81	.43	.10	**6.60** ***	2.77	.43	.10	**6.50** ***	2.56	.42	.10	**6.03** ***	2.08	.40	.08	**5.26** ***
BFI-10 - Agreeableness					-.43	.21	-.03	**-2.09** *	-.39	.21	-.03	-1.88	-.37	.21	-.03	-1.79	-.39	.21	-.03	-1.91	-.23	.19	-.02	-1.18
BFI-10 - Conscientiousness					-1.55	.21	-.12	**-7.44** ***	-1.39	.22	-.11	**-6.48** ***	-1.37	.21	-.11	**-6.38** ***	-1.27	.22	-.10	**-5.94** ***	-1.00	.20	-.08	**-5.02** ***
BFI-10 - Emotional Stability					.03	.17	.00	0.19	.23	.19	.02	1.18	.22	.19	.02	1.16	.24	.19	.02	1.26	.30	.18	.03	1.68
BFI-10 - Extraversion					.19	.19	.01	1.03	.22	.19	.02	1.18	.21	.19	.02	1.14	.21	.18	.02	1.15	.14	.17	.01	0.83
BFI-10 - Openness					.72	.17	.07	**4.15** ***	.68	.17	.06	**3.88** ***	.70	.17	.06	**4.02** ***	.69	.17	.06	**3.97** ***	.45	.16	.04	**2.79** **
GAD-7									-.03	.11	-.01	-0.26	-.10	.11	-.02	-0.76	-.06	.11	-.01	-0.56	-.00	.10	-.00	-0.03
PHQ-9									.35	.11	.08	**3.24** **	.33	.11	.07	**3.02** *	.37	.11	.08	**3.40** ***	.34	.10	.08	**3.34** ***
PSS									-.01	.06	-.01	-0.20	-.05	.07	-.02	0.78	-.02	.06	-.01	-0.27	-.02	.06	-.01	-0.31
Fear for COVID-19													.07	.02	.06	**3.74** ***	.07	.02	.06	**3.77** ***	.01	.02	.01	0.34
Economic Stability																	.09	.01	.11	**6.59** ***	.03	.01	.04	**2.30** *
Self-justifications																					.34	.01	.37	**23.89** ***
*R* ^*2*^	.07				.09				.10				.10				.11				.23			
*R* ^*2*^ *Change*	.07				.02				.00				.00				.01				.12			
*F for change in R* ^*2*^	76.93.53***				16.72***				5.55***				13.98***				43.37***				570.47***			

*Note*. BFI-10 = The Big Five Inventory 10-item. GAD-7 = Generalized anxiety disorder. PHQ-9 = Patient Health Questionnaire. PSS = Perceived Stress Scale.

*p < .05,

**p < .01,

***p < .001.

## Discussion

The present study aimed to examine changes in consumer behavior and their psychological antecedents during the lockdown period due to the COVID-19 pandemic. We were specifically interested in separating necessity and non-necessity products since previous studies suggested that such a distinction is helpful to better understand consumer behavior[18, 74]. First, our results indicated a 61% increase in spending levels during the first week of the lockdown, compared to the average expenses before the health crisis. Furthermore, spending levels were differently increased for buying products framed as necessities (91%) and non-necessities (36%). Second, we examined consumer behavior through Necessities and Non-necessities scales, which included measures related to the psychological need of buying, the specific aspects of the purchase experience (e.g., impulsiveness, perceived utility, satisfaction), and the number of products purchased. Our results highlighted that changes in consumer behavior were positively associated with changes in spending levels during the COVID-19 emergency.

Finally, we focused on psychological factors that can explain these changes in consumer behavior. In this context, our hypothesis about the role of the identified psychological factors in predicting consumer behavior during COVID-19 was supported. Also, our findings confirmed the importance of separating necessities from non-necessities products, as we found that they had different psychological antecedents. Regarding the investigation on spending levels, our findings are in line with sales data reporting that, during the COVID-19 pandemic, consumer priorities have become more centered on necessities, including food, hygiene, and cleaning products[7, 62]. Therefore, the present study confirmed the greater tendency to buy necessities products during the COVID-19 pandemic. It is noteworthy to mention that our sample also reported an increase in spending levels related to non-necessities products. These data can be explained by referring to previous research that considered increases in non-necessities spending levels to respond to the hedonistic pursuit of freedom, defying boredom, restoring the sense of self, and compensatory mechanism, to alleviate negative psychological states[16, 32, 34, 37, 44, 75]. However, as highlighted in the study by Forbes and colleagues[76] these hedonic needs and compensatory mechanisms can have a different impact during or in the aftermath of a crisis. In addition, the authors highlighted that the consumption of non-necessities products increased, as a way of coping to alleviate negative psychological states, particularly in the short term after a natural disaster. According to these results, a recent study conducted during the COVID-19 pandemic suggested that some factors, such as the degree of perceived threat, may vary during the COVID-19 pandemic, thus, having a different impact on consumer behavior[77]. Therefore, future research could delve into the analysis of changes in consumer behavior over time in relation to the different phases of the COVID-19 pandemic.

Regarding our investigation of consumer behavior’s antecedent psychological factors, we found partly different antecedents for necessities and non-necessities. Regarding demographic effects, in the present study, we found that men were more oriented in terms of needs and feelings toward non-necessities than women. A possible explanation could consider the context of the COVID-19, whereas the lockdown has imposed the closure of physical stores. In this context, it could be appropriate to refer to those studies that found several gender differences between consumer e-commerce adoption and purchase decision making. Specifically, research has shown that men and women have different psychological pre-disposition of web-based purchases, with men having more positive attitudes toward online shopping[78, 79]. Furthermore, a study conducted during COVID-19 showed that women spent more time on necessities such as childcare and chores compared to men[80]. Regarding age differences, we found that younger people were more oriented toward non-necessities products. A study conducted in Italy during the COVID-19 pandemic highlighted that older adults showed lower negative emotions than younger adults[73, 81, 82]. In this view, it is possible that lower emotional antecedents, such as depressive states, lowered the need to buy non-necessities for more aged people. Another study conducted during the COVID-19 pandemic showed that older adults, aged 56 to 75, had significantly reduced the purchase of non-necessities goods compared to younger people[83]. Furthermore, considering the closure of physical stores, it is possible that younger people were more able and got used to buy a broader range of non-necessities products by e-commerce. However, it is important to note that we excluded in the present study people aged over 65. We also found a positive effect of income on necessities. A possible explanation is that people more stable from an economic point of view were more oriented to feel the need to buy products. However, surprisingly we did not find this effect for non-necessities. Finally, we found a positive effect of education on non-necessities. This data is congruent with another study conducted during the COVID-19 pandemic, showing that people with higher education (e.g., bachelor’s degrees and graduate or professional degrees) tended to buy an unusual amount of goods than people with lower education[84].Furthermore, another study highlighted that during COVID-19 pandemic entertainment and outdoor expenses significantly varied across different education groups[85]. Considering the present results, further studies should better investigate the impact of socio-demographic factors on the need to purchase necessities and non-necessities during health emergency and natural disaster.

Furthermore, after accounting for control variables (gender, age, income brackets, and education), consumer behavior toward necessities was explained by personality traits (openness), negative emotions (anxiety and COVID- related fear), perception of economic stability, and self-justifications. On the other side, consumer behavior toward non-necessities was explained by conscientiousness, openness, depression, perceived economic stability, and self-justifications.

Present findings showed that negative feelings have a considerable role in predicting changes in consumer behavior related to necessities products. This result is consistent with previous literature showing that, during a health crisis, fear and anxiety are developed from perceived feelings of insecurity and instability[30]. To reduce these negative feelings, people tend to focus on aspects and behaviors that can help them regain control and certainty, such as buying[86]. Therefore, changes in consumer behavior could be explained as a remedial response to reduce fear and anxiety related to the COVID-19 emergency. According to our hypothesis, present findings indicated that fear and anxiety play an important role in predicting changes in consumer behavior related to necessities. In contrast, no significant effects were found on non-necessities. A possible explanation for this remarkable difference can be provided by research in survival psychology, which highlighted that individuals might undergo behavioral changes during events such as natural disasters or health crises, including herd behavior, panic buying, changes in purchasing habits, and decision making[8, 76]. Following these changes, individuals can be more engaged in behaviors that are necessary for survival[26, 87]. In this view, COVID-related fear and anxiety could lead individuals to feel the need to buy necessities products useful for daily survival.

Stress is another factor suggested to differently affect changes in consumer behavior toward necessities and non-necessities[18]. It is noticeable that consumers experiencing stressful situations may show increased spending behavior, explicitly directed toward products that the consumer perceives to be necessities and that allow for control in an otherwise uncontrollable environment[18]. Our results partly support this position, showing that stress has a specific role in predicting changes in consumer behavior related to necessities but not to non-necessities. However, the role of stress was no longer significant when fear was entered in the regression model. Noteworthy, we focused on fear for COVID-19, therefore, it is possible that in such an exceptionally unprecedented situation, fear had a prominent role compared to stress. Moreover, previous literature shows that the relationship between fear and consumer behavior increases as the type of fear measured becomes more specific[88]. In this sense, further studies could delve into the relationship between fear and stress in relation to consumer behavior.

Notably, past studies had found a relationship between depressive states and consumer behavior, suggesting that changes in consumer behavior can represent self-protective behaviors to manage negative affective states[37]. The role of depression was highlighted by our results in respect to consumer behavior only related to non-necessities. Therefore, conversely to the study conducted in the UK and Ireland during the COVID-19 pandemic by Bentall et colleagues (2021), we did not find a relationship between depression and buying necessities. It is important to note that we described non-necessities products as “products for fun or entertainment”. In our opinion, people with higher levels of depressive symptoms may feel a greater need for this kind of product. Thus, people were drawn more toward this category of purchases because it was better suited to satisfy compensatory strategies to improve their negative emotional states. However, future studies are required to investigate this possibility and deepen the relationship between depressive states and the need to buy necessities and non-necessities. Furthermore, considering that depressive mood can be related to severe dysfunctional aspects of consumer behavior, such as impulsivity and compulsivity, future clinical studies should further investigate this relationship.

Furthermore, based on the limited and contrasting literature on this topic, we considered the role of personality traits. As suggested by previous studies, conscientiousness and openness were found to be associated with consumer behavior[89–91]. Interestingly, we found that personality traits were more relevant in consumer behavior toward non-necessities than necessities products. Only openness had a role in (negatively) predicting consumer behavior toward necessities, whereas conscientiousness (negatively) and openness (positively) predicted consumer behavior toward non-necessities. Unexpectedly, we found that people with a high level of openness showed high scores in consumer behavior toward non-necessities but low scores in necessities products. We speculated that individuals with higher levels of openness, which are more inclined to develop interests and hobbies[92], might have experienced a higher need to purchase non-necessities products during the lockdown. On the other hand, individuals with lower scores of openness, which tend to prefer familiar routines to new experiences and have a narrower range of interests, might have been more focused on purchasing necessity products. However, further studies should investigate the different roles of openness on necessities vs non-necessities consumer behavior. Globally, we acknowledge that the specific role and directions of these different personality traits on consumer behavior toward necessities and non-necessities is still an unexplored question, fully deserving of further investigations.

Finally, in both regression models, perceived economic stability and self-justifications predicted changes in consumer behavior. It comes as no surprise that individuals who perceived themselves and their family as more economically stable were prone to spend more in both products categories, necessities and non-necessities [52, 53]. More intriguing, we found that the self-justifications that consumers adopted to motivate their purchases were a strong predictor of consumer behavior, especially in relation to non-necessities, where it explained the largest amount of variance (12%). Therefore, our hypothesis on the greater impact of self-justifications strategies on non-necessities compared to necessities was confirmed. Non-necessities, framed as products for fun or entertainment, seem more suited to satisfy that pursuit of freedom and the need to defy boredom that people increasingly experienced during the COVID-19 pandemic[48]. Therefore, we confirmed that the hedonistic attitude is an important predictor of consumer behavior during the COVID-19 pandemic. This result supported and extended previous literature showing that, during a crisis, changes in consumer behavior are related to self-justifications and rationalizations that people formulate to feel right in making their purchases, including the pursuit of freedom and the reduction of boredom[11, 48]. Companies and markets can acknowledge this process and use it to develop new marketing strategies to meet consumers’ actual needs, feelings, and motivation to purchase during the COVID-19 emergency[12]. On the one hand, satisfying these needs could support and favor well-being and the positive sense of self, which are essentially sought by the consumer developing such self-justification strategies[17]. On the other hand, focusing on strategies that consider these psychological self-justifications could be a winning marketing strategy for increasing sales, contributing to the economic recovery after the COVID-19 outbreak[13].

## Conclusion

The results of the present study highlighted that the COVID-19 pandemic had a considerable impact on consumer behavior. In our sample, this impact resulted in increased spending levels accompanied by an increase in the psychological need to purchase both necessities and non-necessities products. Furthermore, our findings demonstrated that several psychological factors predicted these changes in consumer behavior. Notably, consumer behavior respectively toward necessities and non-necessities differed on some psychological predictors.

Some limits of the current study need to be acknowledged. First, we studied consumer behavior from a broad perspective on a non-clinical sample, therefore we did not include dysfunctional aspects related to consumer behavior, such as impulsivity and compulsivity buying and hoarding behavior, which the emergency may elicit. Hence, in relation to the COVID-19 pandemic, it would be interesting to integrate our results with investigations of dysfunctional aspects of consumer behavior. Furthermore, since the unique opportunity to study psychological factors and consumer behavior during this unprecedented period, we adopted an integrative approach to consider the impact of several psychological factors at once, obtaining one of the first overviews of consumer behavior during the COVID-19 pandemic. However, combining all these psychological factors could have led to an aggregation bias[93], which could have masked the specific roles of each of the individual factors influencing consumer behavior. Therefore, future studies could adopt a more fine-grained approach to disentangle the role of each factor. Another limit is that we collected data during the initial stage of the COVID-19 outbreak in Italy. Notably, we reasoned that focusing on the very first period of the lockdown would likely allow us to capture the greater shift in consumer behavior, thus offering compelling evidence on the first impact of the pandemic on consumers. Nevertheless, it is likely that consumer behavior will undergo further changes in the longer term. Hence, future studies should investigate the evolution of consumer behaviors in relation to the development of the pandemic. Indeed, it is likely that when the “sense of urgency” and the negative affective reaction to the emergency will decrease, also the need for buying and purchases preferences would change. Furthermore, since we asked participants to estimate their weekly expenditures before and during the COVID-19 pandemic, it is important to keep in mind that our study focused on the people’s perception of changes in expenses. We did not know how much reliable these estimations were, and it is possible that objective assessment of change in the amount of money spent before and during the pandemic diverge from subjective views. In the present study, we focused on individual internal factors that could influence consumer behavior. However, other external factors, including the lockdown restrictions as the closure of physical stores, had certainly had a further impact on consumer behavior. Notwithstanding these limitations, this study represents one of the first attempts to examine changes in consumer behavior during the COVID-19 pandemic from a behavioral economic perspective, providing a thorough analysis of the psychological factors driving changes in consumer behavior, with a direct link to previous psychological research in consumer behavior. Furthermore, our results provided new evidence on the role of psychological factors influencing necessities and non-necessities spending and extended our knowledge of the antecedents of consumer behavior changes during the unprecedented health crisis we are experiencing.

In conclusion, the present study, by shedding new light on changes in people’s behavior due to the pandemic, fits into the growing body of research which helps increase economic and psychological preparedness in the face of future health emergencies.

## Supporting information

S1 Table Pattern matrix of the PCA for the questionnaire on consumer behavior during the COVID-19 pandemic. (DOCX)

S2 Table PCA for the “Perceived economic stability” questionnaire. (DOCX)
