# Supplementary material for: Psychological factors and consumer behavior during the COVID-19 pandemic
Source: PLoS One. 2021 Aug 16;16(8):e0256095. doi: 10.1371/journal.pone.0256095 (PMC8366984; doi:10.1371/journal.pone.0256095)
Supplement: S1 Table — (DOCX) [file pone.0256095.s001.docx]

| **S1 Table. Pattern matrix of the PCA for the "Consumer behavior during COVID-19" questionnaire** | | | | | | | |
| --- | --- | --- | --- | --- | --- | --- | --- |
| **Scale** | **Item** | **Factor loadings** | | | | | |
|  |  | **A** | **B** | **C** | **D** | **E** | **F** |
| **A. Necessities** | 1. I have felt the need to buy larger amounts of health and safety products (e.g., alcohol-based hand sanitizer, gloves, face masks) | .72 |  |  |  |  |  |
|  | 2. I believe I impulsively bought Necessities products | .69 |  |  |  |  |  |
|  | 3. I have felt the need to buy larger amounts of Necessities products (e.g., food, health and wellness products, personal hygiene products, house cleaning products) compared to before | .66 |  |  |  |  |  |
|  | 4. If you do not already own health and safety products, how much more would you be willing to spend to acquire these products (e.g., alcohol-based hand sanitizer, gloves, face masks)? | .65 |  |  |  |  |  |
|  | 5. I felt the need to buy products that I did not need before | .52 |  |  |  |  |  |
| **B. Non-necessities** | 1. At the time of purchase, how useful did you think Non-necessities products would be? |  | .83 |  |  |  |  |
|  | 2. At this time, how useful do you think the Non-necessities products that you purchased are? |  | .82 |  |  |  |  |
|  | 3. Have you bought any products that are considered Non-necessities? |  | .69 |  |  |  |  |
|  | 4. I have felt the need to buy larger amounts of Non-necessities products (e.g., products for fun and entertainment) compared to before |  | .67 |  |  |  |  |
| **C. Self-justifications** | 1. Making purchases makes me feel better |  |  | .75 |  |  |  |
|  | 2.Considering that I am restricted to home isolation, I want to enjoy the purchases that I have made |  |  | .72 |  |  |  |
|  | 3. Considering that I am saving money by not going out, I can afford to make a new purchase |  |  | .72 |  |  |  |
|  | 4. I am happy with the purchases that I have made |  |  | .63 |  | . |  |
| **D. Spending habits** | 1. Before COVID-19 emergency, how much do you think you were spending weekly for Necessities products? |  |  |  | .82 |  |  |
|  | 2. During COVID-19 emergency, how much do you think you have spent weekly for Necessities products? |  |  |  | .75 |  |  |
|  | 3. Before COVID-19 emergency, how much do you think you were spending weekly for Non-necessities products? |  |  |  | .73 |  |  |
|  | 4. During COVID-19 emergency, how much do you think you have spent weekly for Non-necessities products? | *.32* |  |  | .59 |  |  |
| **E. Choice strategies*** | 1. I took into consideration the quality of the products that I bought |  |  |  |  | .70 |  |
|  | 2. I think I have accurately selected and chosen the products that I bought |  |  |  |  | .68 |  |
|  | 3. I took into consideration the price of the products that I bought |  |  |  |  | .59 | *.32* |
|  | 4. It is important to save money |  |  |  |  | .44 |  |
| **F. Information*** | 1. I was informed by product reviews, advertisements, and commercials about the products that I bought |  |  |  |  |  | .75 |
|  | 2. I have consulted with other people when choosing the right products to purchase |  |  |  |  |  | .66 |
|  | 3. I chose to buy products based on the brand |  |  |  |  |  | .38 |
| **Items excluded for  cross-loadings** | I believe I impulsively bought Non-necessities products | .44 | *.34* | *.21* |  | *-.26* | *.21* |
|  | I could need this product in the future, but I do not know if it will be possible to purchase it in the next few weeks |  |  | *.34* | .51 |  |  |
|  | I chose to buy products that I used to buy in the past |  |  |  |  | .60 | *-.34* |
| *Note.* Items referred to the COVID-19 emergency period compared to before COVID-19 outbreak.  Factor loadings with values higher than .20 but lower than .40 are reported in italics.  * Scales not analyzed in the current study. | | | | | | | |
