# Supplementary material for: Psychological factors and consumer behavior during the COVID-19 pandemic
Source: PLoS One. 2021 Aug 16;16(8):e0256095. doi: 10.1371/journal.pone.0256095 (PMC8366984; doi:10.1371/journal.pone.0256095)
Supplement: S2 Table — (DOCX) [file pone.0256095.s002.docx]

| **S2 Table. PCA for the "Perceived economic stability" questionnaire** | |
| --- | --- |
| **Item** | **Factor loadings** |
| Before the COVID-19 crisis, I considered my family and I to be financially stable | .87 |
| During the COVID-19 crisis, I considered my family and I to be financially stable | .94 |
| I believe that my family and I will continue to be financially stable when the COVID-19 crisis is over | .91 |
